# Dengue, Chikungunya, and Zika: The Causes and Threats of Emerging and Re-emerging Arboviral Diseases

**DOI:** 10.7759/cureus.41717

**Published:** 2023-07-11

**Authors:** Suha Soni, Vikram Jeet Singh Gill, Anusheel, Jugraj Singh, Jayksh Chhabra, Gurparam Jeet Singh Gill, Rupinder Bakshi

**Affiliations:** 1 Department of Public Health Sciences, University of Texas Health Science Center at Houston, Houston, USA; 2 Department of Internal Medicine, Baylor College of Medicine, Houston, USA; 3 Department of Internal Medicine, Shanti Gopal Hospital, Ghaziabad, IND; 4 Department of Internal Medicine, Punjab Institute of Medical Sciences, Jalandhar, IND; 5 Department of Internal Medicine, California Institute of Behavioral Neurosciences and Psychology, Fairfield, USA; 6 Department of Otolaryngology, Head and Neck Surgery, Adesh Medical College and Hospital, Ambala, IND; 7 Department of Microbiology, Government Medical College, Patiala, IND

**Keywords:** mosquito vector, vector borne diseases, sero-surveillance, zika infection, chikungunya encephalitis, dengue shock syndrome (dss), dengue hemorrhagic fever (dhf), dengue fever (df), dengue thrombocytopenia, dengue virus infection

## Abstract

The recent emergence and re-emergence of viral infections transmitted by vectors, Zika, chikungunya, dengue, and others, is a cause for international concern. Here, we provide a summary of the current understanding of the transmission, clinical features, diagnosis, global burden, and the likelihood of future epidemics by these viruses. Arboviruses transmitted by mosquitoes are challenging to diagnose and can have surprising clinical complications. Dengue, chikungunya, and Zika are the most important diseases caused by arboviruses worldwide, especially in tropical and subtropical regions. These are transmitted to humans by day-biting *Aedes aegypti* and *Aedes albopictus* mosquitoes. In India, the increase in the incidence of dengue and chikungunya cases is primarily linked to the dissemination of *Aedes aegypti*. A rapid and accurate diagnosis is paramount for effectively controlling dengue outbreaks. As there is no vaccination or specific treatment available for these viruses, vector control is the only comprehensive solution available.

## Introduction and background

Among the most neglected human pathogens, arboviruses (arthropod-borne viruses) cause the majority of morbidity and mortality in tropical and subtropical regions since mosquitoes are abundant in these areas [1]. It has been observed in the past few years that some arboviruses, such as dengue (DENV), chikungunya (CHIKV), and Zika (ZIKV), are extending their geographical range, ultimately causing regional transmission and causing significant outbreaks in almost all continents, especially in the temperate zones [1,2]. Dengue fever (DF) is the most important mosquito-borne viral infection responsible for high human mortality. It is caused by the DENV, which belongs to the Flaviviridae family. It is most prevalent in tropical and subtropical regions [3].

The frequency of dengue has substantially increased in recent decades, according to the WHO, with cases reported to the organization rising from 505,430 cases in 2000 to 5.2 million cases in 2019 [4]. Dengue cases are underreported since many of them are asymptomatic, moderate, and self-managed. In many situations, other febrile infections are mistakenly identified [5]. According to an estimate, 390 million DENV infections occur yearly across the globe, of which 96 million result in clinical manifestations [6]. Today, 40% of the world's population lives in areas with a high risk of dengue transmission. Dengue is endemic in nearly 100 countries in tropical and subtropical regions [7]. According to WHO estimates, between 50 and 100 million infections occur annually, with 500,000 cases of dengue hemorrhagic fever (DHF) and 22,000 deaths, mostly involving children [8].

The first DF outbreak in India occurred in 1812, and subsequent large outbreaks occurred in 1836, 1906, 1911, 1972, 2005, 2010, and 2015. Since the first dengue pandemic in Kolkata in the 1960s, several dengue outbreaks have been reported often from various locations in India [9]. The illness has recently developed a severe form known as DHF, and outbreaks are happening more frequently [10]. Delhi saw one of the largest dengue epidemics in North India in 1996, which was caused mainly by serotype 2. In contrast, in 2003, dengue serotype 3 was to blame for the outbreak in North India [11]. There has been a significant increase in dengue cases in India over the years: 188 407 (2017), 157 315 (2019), and 193 245 (2021), and the worst affected cities were UP, West Bengal, and Delhi, followed by Punjab [12].

CHIKV was initially identified as an alphavirus in Africa (Tanzania) in 1954. *Aedes aegypti* and *Aedes albopictus* mosquitoes that bite humans during the day are the carriers of both illnesses [13]. Although there were several ZIKV outbreaks in the Pacific Islands between 2007 and 2014, it was not until mid-2015 that this mosquito-borne flavivirus was first identified in Brazil among individuals with a disease similar to DENV. ZIKV gained international attention in late 2015 following increased reports of congenital microcephaly and fetal CNS abnormalities among pregnant women infected with ZIKV in Brazil [14]. Before an epidemic was declared in Rajasthan in 2018, the Ministry of Health and Family Welfare (MoHFW) of the Government of India announced three laboratory-confirmed cases of the ZIKV sickness in the Bapunagar district of Gujarat State's Ahmedabad District on May 15, 2017 [15].

The occurrence of emerging infections and re-emerging infections are influenced by a variety of factors, including human conduct and microorganism adaptation to the myriad of ecological factors (globalization, public health infrastructure, etc) [16]. In addition, most of these factors could be associated with overpopulation, poor sanitation facilities, and increased exposure of humans to microbial-carrying vectors [17].

Understanding the potential interactions on ZIKV, DENV, and CHIKV multiplication in concurrently infected vector mosquitoes is crucial, given that India has known endemic regions for DENV and CHIKV transmission by these *Aedes* mosquitoes [18]. This review offers an update on our knowledge of DENV, CHIKV, and ZIKV pathophysiology, diagnosis, and prophylaxis.

## Review

DENV pathogenicity

DENV usually replicates in mononuclear cells, such as skin dendritic cells, tissue macrophages, hepatocytes, and peripheral blood monocytes [19]. Dendritic cell-specific ICAM3-grabbing non-integrin, a non-specific receptor, allows DENV to infect immature dendritic cells in the skin. Dendritic cells that have been infected mature and go to regional lymph nodes, where the T cells are exposed to viral antigens, starting the cellular and humoral immune responses [20]. DENVs are further replicated in peripheral blood monocytes, hepatocytes, and macrophages in the liver, spleen, and lymph nodes [21]. Although the DEN viruses have close serological relationships, they differ antigenically [20].

DF typically results from the primary or initial infection in non-immune individuals [22]. A new serotype of dengue infection later on results in more severe sickness, like DHF and dengue shock syndrome (DSS). The three main symptoms of DHF/DSS are capillary leakage, abrupt onset of shock, and hemorrhagic diathesis/thrombocytopenia that co-occurs as fever defervescence [23].

**Figure 1:**
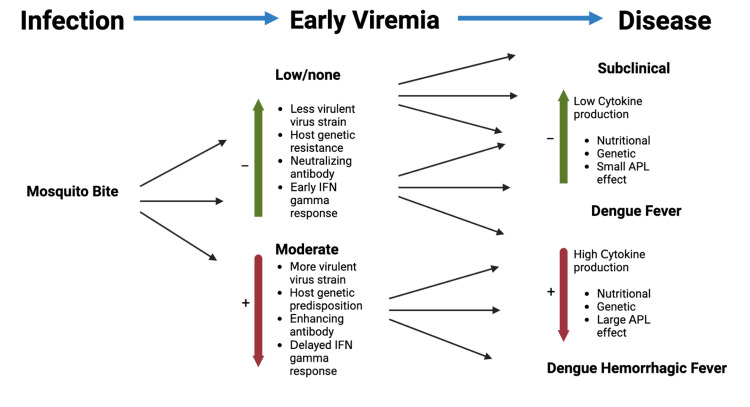
Factors affecting the severity of dengue infection IFN: interferon, APL: altered peptide ligand

Primary infection by one DENV serotype does not protect against infection from another [24]. According to the antibody-dependent enhancement hypothesis, circulating IgG antibodies form complexes with the virus during active infection and promote virus uptake by macrophages where it undergoes replication. This results in a high viral antigen load leading to an exaggerated activation of T cells followed by DHF and DSS. It is characterized by a diminished IgM antibody response, followed by the release of cytokines and vasoactive mediators, which will increase vascular permeability and hemorrhage (Figure 1). This will result in disseminated intravascular coagulation, followed by vascular collapse, which may lead to the patient's death [21].

Clinical manifestations of dengue

*Dengue Fever*

DF involves primary and secondary infections most frequently encountered in adults and older children. The onset of symptoms is marked by a biphasic, high-grade fever lasting from three days to one week [25]. It is also associated with severe headaches (mostly retro bulbar), muscle cramps, joint pain, diarrhea, vomiting, and cutaneous rash. The incubation period for DENV is four to seven days (range 3-14 days). The DF stage is seen mainly in the primary infection but may also occur following the initial secondary infection. Clinically, differentiating DF from other viral diseases is challenging; hence, it often remains undiagnosed [10].

Dengue Hemorrhagic Fever

DHF may occur during a primary infection due to maternally attained dengue antibodies in infants but is frequently followed by a secondary infection in the case of adults [26]. Hemorrhagic episodes presenting at least one of the following: petechiae, purpura, ecchymosis, nose/gum bleeding and mucosal bleeding, hematemesis, and/or melena form the diagnostic criteria of DHF [25].

The bleeding in DHF is linked with various factors, i.e., platelet deficiency, defects in the blood coagulation pathways, and vasculopathy [27]. Decreased platelet production and increased platelet dysfunction and destruction result in thrombocytopenia. These dysfunctional platelets cause blood vessel fragility and, thus, result in bleeding [28].

There are three stages in the course of DHF: fever, plasma leak, and the convalescent phase [29]. In the first stage, the patient develops a rash and experiences hemorrhage. The febrile stage is approximately two to seven days, and the convalescent phase, or the plasma leakage phase, follows the first phase. Plasma leakage can lead to hypovolemic shock with bradycardia, cyanosis, hepatomegaly, pleural and pericardial effusions, and ascites [10].

Dengue Shock Syndrome

DHF, complicated by an unstable pulse, narrow pulse pressure, cold skin, restlessness, and cyanosis around the mouth, is defined as DSS. Hypovolemic shock, multi-organ damage, and consumption coagulopathy contribute to a high mortality rate in DSS. Usually, the shock persists for a short span, and the patient promptly recovers with supportive therapy [25].

Differential diagnosis in a patient presenting with fever and rash similar to dengue 

When a patient presents with a fever and rash similar to those seen in DF, a broad differential diagnosis is considered. The various clinical conditions that resemble the febrile phase of dengue infection are listed in Table 1 [30].

**Table 1:** Conditions that mimic dengue-like infection in the febrile phase HIV: human immunodeficiency virus

General presenting symptoms of the diseases	Possible diagnosis
Flu-like syndromes	Influenza, measles, chikungunya, infectious mononucleosis, HIV seroconversion illness
With rash	Measles, scarlet fever, rubella, meningococcal infection, chikungunya, drug reactions
Diarrhoeal diseases	Rotavirus and other enteric infections
Illnesses with neurological manifestations	Meningoencephalitis febrile seizures

CHIKV pathogenicity

CHIKV belongs to the genus alphavirus, the family Togaviridae. It is an enveloped virus with a positive-strand RNA virus. It encodes four nonstructural proteins (nsP1 to nsP4) and five structural proteins (C-E3-E2-6K-E1). CHIKV could be detected in connective tissue, muscle, joint, skin fibroblast, and the CNS [31]. The transmission of CHIKV is in two cycles: urban (human to mosquito to human) and sylvatic (animal to mosquito to human) [32]. The route of infection in humans is different, compared to other arboviruses, with certain cell types being particularly susceptible to infection. These cells include human epithelial and endothelial cells, fibroblasts, and monocyte-derived macrophages, whereas primary lymphocytes, monocytes, and monocyte-derived dendritic cells did not demonstrate CHIKV replication [33]. The immune response to CHIKV infection has been partly described, but considerable portions of it remain unrecognized.

Clinical manifestations of chikungunya

The incubation period is 1 to 12 days (range 3-7 days) [31].

Acute Stage

The acute stage is the first 10 days after disease onset. The most common symptoms are high-grade fever, arthralgia, back pain, and headache. This stage may be associated with fatigue, anorexia, myalgia, nausea, and vomiting. Peripheral joints, i.e., interphalangeal joints, wrists, and ankle joints, are frequently involved [34]. They are swollen and painful and can be treated with non-steroidal anti-inflammatory drugs. Aspirin should be avoided as it can cause bleeding problems. After 10-15 days, the symptoms subside [13].

Chronic Stage

After the acute stage, there is a relapse of inflammatory symptoms in CHIKV patients. There is long-lasting rheumatism, especially if there has a high viral load of CHIKV in the acute stage. Within three months, patients have a relapse of joint pain in distal joints and inflammation of tendons. Many patients may have carpal or tarsal tunnel syndrome [35].

ZIKV pathogenicity

ZIKV is unique in its replication as most flavivirus replicates in the cell cytoplasm, whereas ZIKV multiplies in the infected cell nuclei [36]. The ZIKV pathogenesis is still under research, and less is known about its pathogenesis. Still, most mosquito-borne flaviviruses are known to replicate near the bite site in dendritic cells. The virus then spreads through lymph nodes, followed by the bloodstream [37]. There is insufficient data regarding the incubation period, its appearance in body fluids, and the duration it is present in the body. It can be detected as early as the onset of the illness begins and even after 11 days of the onset of the disease in human blood [38].

Clinical features of ZIKV infection

Symptoms of infection with the virus begin with mild headache followed by a maculopapular rash (neck, face, trunk, and upper arms, and spread to palms and soles), fever, malaise, conjunctivitis, and joint pains. It also causes microcephaly in newborn babies through mother-to-child transmission and neurologic conditions in infected adults, including Guillain-Barré syndrome [36].

Management of DENV, CHIKV, and ZIKV infection

For now, no vaccine or antiviral medication can stop the spread of DENV, CHIKV, or ZIV. Rest, water (to prevent dehydration), and paracetamol (acetaminophen) are the mainstays of symptomatic treatment [36]. Due to the risk of bleeding or hemorrhages, aspirin, and other non-steroidal anti-inflammatory medications should be taken with caution if the patient also has a DENV infection; therefore, before taking this medication, dengue should be ruled out [39].

The foundation of treatment is judicious fluid administration during the critical infection period. Normal saline, Ringer's lactate, 5% glucose diluted 1:2 or 1:1 in normal saline, plasma, plasma replacements, or 5% albumin are typically the fluids that are administered [38].

WHO guidelines summarize the following principles of fluid therapy

Supplemental oral fluid intake must be as extensive as possible. However, intravenous fluid administration is required when the patient cannot consume fluids orally due to shock, acute vomiting, or prostration.

We use crystalloids such as 0.9% saline as the first choice in intravenous fluids. The second-line treatments are mainly used for hypotensive conditions and are non-responsive to intravenous crystalloids or colloids like dextran infusions. There should be a serious concern for bleeding if the patient's platelet levels are still low and in the critical stage. Fresh whole blood transfusions are the best treatment option for suspected bleeding cases [40].

Laboratory diagnosis of DENV, CHIKV, and ZIKV infection

Laboratory diagnosis of dengue infection is difficult because of a wide range of clinical presentations, ranging from mild febrile illness to several severe syndromes [41]. Due to the presence of pre-existing antibodies and the phenomenon of original antigenic sin (during sequential flavivirus infections, B-cell clones responding to the first infection synthesize antibodies with higher affinity for the first infecting virus than for the second infecting virus), multiple and sequential flavivirus infections make differential diagnosis difficult in areas where two or more flaviviruses are circulating [18].

A probable dengue infection is indicated by IgM or high IgG levels in acute serum taken from a suspected dengue case [42]. Serological testing, i.e., Mac-ELISA (IgM) and NS1 antigen detection for DF and IgM Mac-ELISA for chikungunya, is done routinely in a tertiary-care center in India [43].

**Figure 2:**
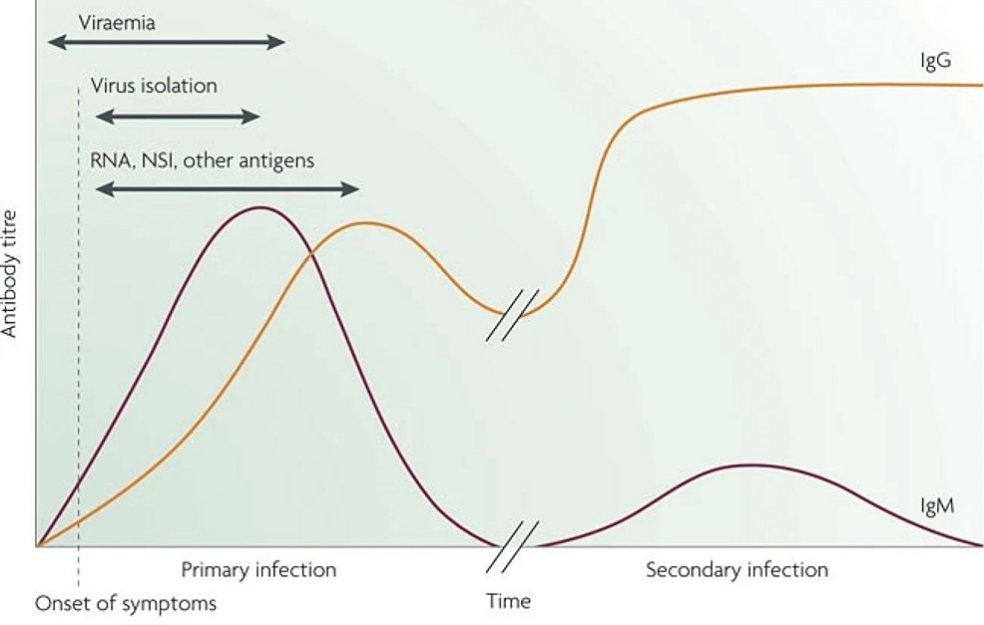
DENV, antigen, and antibody responses used in diagnosis

Serological tests are performed on samples five to six days after the onset of symptoms of ZIKV acute samples [44]. The preferred test is the acute-phase serum samples' reverse transcription polymerase chain reaction test (RT-PCR). ELISA can detect IgM immunoglobulins to ZIKV as early as three days after illness onset, but commercial kits are unavailable in India [45].

However, virus isolation and molecular techniques provide the most specific test result among the methods available for DENV, CHIKV, and ZIKV diagnosis, and they provide good evidence of infection in the acute phase [46]. However, facilities supporting viral culture are only sometimes available in some tertiary care hospitals.

Prevention from arboviral diseases

It is tough to have a single effective method of prevention of flavivirus infection in the affected areas such as the tropics. However, infection risk can be decreased effectively by understanding the vector's biological behavior and feeding habits. Based on this understanding, simple precautions can be taken to reduce exposure to infective mosquito bites. Female *Aedes aegypti* mosquitoes prefer to feed indoors, with peak biting activity occurring two to three hours after daybreak and three to four hours before nightfall [4]. Precautions, therefore, include wearing protective clothing, using mosquito repellent, and avoiding the collection of clean water in houses, plants, and coolers can prevent the spread of mosquitoes. Early diagnosis, treatment, and prompt preventive measures can prevent the spread of the DENV, CHIKV, and ZIKV [46].

## Conclusions

We discussed in the paper three important arboviral diseases, i.e., dengue, chikungunya, and Zika. All three conditions have affected a significant chunk of the world’s population, especially those living in resource-deficient parts of the world. Therefore, it becomes imperative for clinicians and healthcare workers worldwide to know the pathogenicity, clinical manifestations, diagnosis, and management of these dreaded viral infections. The articles mention the importance of effective prevention and mitigation strategies for arboviral diseases. Apart from the current practice of insect-behavior-based prevention strategies, potential future strategies such as the creation of vaccines, antiviral medication regime development, and innovative ways to control the vectors can be worked on.
